# The Burnout of Nurses in Intensive Care Units and the Impact of the Pandemic of SARS-CoV-2: Protocol of a Scoping Review

**DOI:** 10.3390/nursrep12030065

**Published:** 2022-09-05

**Authors:** Andreia Lima, Maria Teresa Moreira, Carla Fernandes, Margarida Ferreira, Joana Teixeira, Vítor Parola, Adriana Coelho

**Affiliations:** 1 Health Sciences School—Fernando Pessoa, CINTESIS, 4200-253 Porto, Portugal; 2 Nursing School of Porto, CINTESIS, 4200-072 Porto, Portugal; 3 Health Sciences School Jean Piaget Vila Nova de Gaia, CINTESIS, 4405-678 Vila Nova de Gaia, Portugal; 4 Health Sciences School—Fernando Pessoa, 4200-253 Porto, Portugal; 5 The Health Sciences Research Unit: Nursing (UICISA:E), Nursing School of Coimbra (ESEnfC), 3004-011 Coimbra, Portugal

**Keywords:** burnout, professional, nurses, intensive care units, SARS-CoV-2, review

## Abstract

**Background:** The SARS-CoV-2 pandemic has brought multiple challenges for health institutions and their professionals. The requirement of this disease forced nurses to confront organizational and clinical challenges to maintain the quality standards of care they provide. These requirements may have contributed to increased burnout symptoms. This study aims to map the scientific evidence related to nurses’ burnout in intensive care units. **Methods**: A scoping review will be conducted according to the Joanna Briggs Institute methodology. Relevant databases will be used as well as grey literature, where the following words will be used: burnout, nurses, intensive care units and SARS-CoV-2. **Results**: This scoping review will include all types of studies—quantitative, qualitative and mixed—and all types of reviews that focus on the objective of this review. **Conclusions**: It is vital to determine the impact of the burnout caused by the pandemic of SARS-CoV-2 to assess amending measures of risk and protection factors. This will help in the implementation of guidelines according to the available evidence. Additionally, this will help to improve the skills of these professionals as well as to reduce their emotional and physical exhaustion. This protocol is registered with the Open Science Framework.

## 1. Introduction

SARS-CoV-2 is a virus responsible for the coronavirus pandemic 2019 (COVID-19), which has consigned humanity to fighting a serious public health problem caused by a severe respiratory syndrome among both healthy people and people with chronic health problems. It all started in China in December 2019, quickly spread to Asian countries such as Thailand, Japan, South Korea and Singapore and then moved to Europe and other continents. The World Health Organization (WHO) then declared a public health emergency of international relevance, dated 30 January 2020, and a pandemic on 11 March 2020 [1].

Around 80% of patients in 2020 had respiratory infections and mild pneumonia, but the most severe cases affected people with chronic diseases and the elderly. The need for hospitalization and even the use of mechanical ventilators brought stress to an already challenging and crowded hospital [2]. About 6 to 10% of those infected were sent to intensive care units [3] due to the severity of their health situation. These clinical issues led to the urgency to increase the number of technical, technological and healthcare professionals and reformulate the institutions’ infrastructure [4,5].

All the demands arising from the environmental and situational stress caused by the need to provide immediate responses to this unknown situation, along with the need to implement infection control measures, constituted risk factors for the development of mental symptoms in the general population such as anxiety and depression [6,7], and nurses were no exception.

Due to the demands resulting from the pandemic situation, many organizational, political, social and even legal changes were necessary to avoid further and major consequences of the disease and to control the problem caused by it, and they lasted for two years. These changes inevitably influenced the health of the care professionals, and with nurses, these numbers were considerably larger [8,9].

Intensive care units are considered services that are creators of professional stress due to the severity and complexity of the situations presented there [10]. According to some studies, the pandemic triggered all factors that increase pressure and exhaustion in those professionals [9,11].

According to Maslach and Leiter, burnout results from overwork, prolonged work periods, low salaries, interprofessional conflicts, work overload, a lack of human and material resources and even professional dissatisfaction [12,13]. Several studies relate the high levels of burnout syndrome among nurses to their work-related placement, and these levels are higher among those working in intensive care units [11,12,13,14,15]. It is also confirmed that these levels are higher for these professionals compared to other members of the health multidisciplinary team [16].

Building strategies aimed at nurses’ health that are affected by work overload are of interest, and the evidence of an increase in burnout during the SARS-CoV-2 pandemic can bring forward protective and preventive measures for future situations of similar or increased severity.

A preliminary literature review was conducted in PROSPERO, MEDLINE, Open Science Framework (OSF), the Cochrane Database of Systematic Reviews and Joanna Briggs Institute (JBI) Evidence Synthesis using the search terms: ICU, Nurses, burnout and COVID-19. This revealed the inexistence of a scoping review on the topic. It was also possible to assess and adapt the keywords for each of the databases later included in this review.

The aim of the present review is therefore to map the scientific evidence related to the burnout of nurses in an intensive care unit and to analyze the prevalence of this syndrome in these nurses during the COVID-19 pandemic.

## 2. Materials and Methods

The protocol of this scoping review follows the guidelines of the Joanna Briggs Institute (JBI) methodology [1,2]. For the final review, the items identified in the reports prepared for the guidance of systematic reviews and the extension of meta-analyses (PRISMA-ScR) will be used [3]. This protocol was registered in the OSF (https://osf.io/8s7a6/ (accessed on 22 April 2022)).

### 2.1. Inclusion Criteria

Based on JBI recommendations and using the mnemonic PCC for scoping reviews, we have selected the following inclusion criteria: concerning the participants, this review will consider studies that include nurses; regarding the concept, this review will include studies addressing the burnout of nurses; concerning the context, this review will consider articles whose study period occurred during the pandemic of SARS-CoV-2 and which involve intensive care units, regardless of the country of the study; regarding the types of sources, this scoping review will consider all study typologies, i.e., quantitative, qualitative and mixed methods, and all kinds of literature reviews.

### 2.2. Search Strategy

Two reviewers developed the search strategy, which was peer-reviewed by the expert third reviewer considering the Peer Review of Electronic Search Strategies (PRESS) checklist. The research strategy will include primary and secondary studies published and not published in the following databases: Medical Literature Analysis and Retrieval System Online (MEDLINE^®^) via PubMed, Cumulative Index to Nursing and Allied Health Literature (CINAHL^®^), LILACS, SCOPUS, PsycINFO and OPEN GREY.

The research strategy recommended by JBI will be implemented [1,2]. A preliminary search was carried out in the MEDLINE (via PubMed) and CINAHL Complete (EBSCOhost) databases to identify the keywords and index terms used in the articles on the subject. Through this research, the search strategy was created for each database, considering its specificities, as shown in Table 1. This survey was conducted on 14 April 2022. The reference lists of all included articles will be reviewed for the possibility of the inclusion of additional articles. After the search, the identified articles will be deposited in the ENDNOTE program, and RAYYAN and duplicates will be removed.

### 2.3. Study Selection

The data will be extracted from the articles to be included in this scoping review by two reviewers independently; both doubts and disagreements will be discussed using a third reviewer according to the peer review of the Electronic Search Strategies (PRESS) checklist [6]. The pilot test will be carried out by two independent reviewers, beginning with analyzing the title/abstract and, later, the full text. For its analyses, 5% of the total research will be used to obtain at least a 75% consensus between reviewers. In the second phase of the study, 2% of the full-text articles will be used to obtain the same level of agreement. Studies that include the corpus of analysis of this review will be obtained through a strategy that includes the identification, selection, eligibility and inclusion of the same conducted by the inclusion criteria and the research limiters [3]. The extracted data will include specific details about the population, concept, context, study method and main evidence relevant to the objective of this review, as shown in Table 2.

A PRISMA-ScR flow diagram will document the selection process [3].

### 2.4. Data Analysis and Presentation

Data will be extracted from the studies included in the review using an extraction tool according to the objectives and research questions of the present review. The tool used will be conducted by the methodology proposed by the Joanna Briggs Institute [3,6], including the relevant information: title, author(s), year of publication, country of origin, type of study, objective(s) and results, as shown in Table 2.

The collected data will be presented in narrative form using a qualitative assessment tool and corresponding coding. This will consider all aspects measured for burnout: emotional exhaustion, depersonalization and personal accomplishment. To map the available evidence and complement the information of each of the articles, a table will include the above and relevant information. The identification, characterization and synthesis of the knowledge that this review will bring will be related to the proposed objective and review question.

## 3. Discussion

This scoping review will gather the information necessary to identify the level of burnout among nurses who worked in an ICU context and its consequences during the pandemic of Sars-CoV-2. This strength will direct the findings to institutional managers to implement preventive measures and promote healthy environments in this specific work context. We also hope to identify research gaps that will guide further studies in this area. This study will thus make it possible to obtain an overview of the preventive measures used, which is of great value in future situation. In fact, the emergence of new viruses can already be seen, which could eventually lead to a new stress on health institutions and it is essential that we are prepared for them [17].

Articles published in the last three years on the subject under study and studies written in English, Portuguese and Spanish will be included in this scoping review. However, the language limitation may be one of the limitations of this study.

This study aims to map the scientific evidence related to nurses’ burnout in intensive care units. Since this is a scoping review, the critical evaluation of the included studies will not be provided; however, if corroborated, the limitations will be reported, as they may be of value for future investigations, particularly for systematic reviews.

## 4. Conclusions

This review is expected to be a starting point for mapping the available scientific evidence on the subject under study, contributing to the prevention of burnout among nurses and implementing new programs to improve the health of professionals and working conditions. In the future, it may also contribute to the prevention of burnout in the workplace in emergencies like the one that occurred with the pandemic of COVID-19.

## Figures and Tables

**Table 1:** Database search strategy and results.

Database: CINAHL Complete (via EBSCO) Filters: last 3 years, English, Portuguese, Spanish, excluding MEDLINE Results: 22 Search strategy (14 April 2022) (TI SARS-CoV-2 OR AB SARS-CoV-2 OR MH SARS-CoV-2 OR TI COVID-19 OR AB COVID-19 OR MH COVID-19) AND (TI burnout OR AB burnout OR MH burnout, professional OR TI exhaustion OR AB exhaustion) AND (TI nurse OR AB nurse OR MH nurses) AND (TI intensive care units OR AB intensive care units OR MH intensive care units OR TI intensive care unit OR AB intensive care unit OR TI ICU OR AB ICU)
Database: Psychology and Behavioral Sciences Collection Filters: last 3 years, English, Portuguese, Spanish Results: 2 Search strategy (14 April 2022) (((DE “PSYCHOLOGICAL burnout”) OR (burnout) OR (exhaustion)) AND ((DE “NURSES”) OR (nurse) OR (nurses) OR (DE “NURSE practitioners”)) AND ((DE “INTENSIVE care units”) OR (intensive care units) OR (intensive care unit) OR (ICU)) AND ((DE “SARS-CoV-2”) OR (SARS-CoV-2) OR (DE “COVID-19”) OR (COVID-19)))
Database: LILACS Filters: last 3 years, English, Portuguese, Spanish Resultados: 10 Estratégia de pesquisa (14 April 2022) (COVID AND burnout AND nurse)
Database: SCOPUS Filters: last 3 years, English, Portuguese, Spanish Results: 208 Search strategy (14 April 2022) ((TITLE-ABS-KEY ((burnout, professional) OR (burnout) OR (exhaustion)) AND TITLE-ABS-KEY ((nurses) OR (nurse) OR (nurses) OR (nurse practitioners)) AND TITLE-ABS-KEY ((intensive care units) OR (intensive care units)) OR (intensive care unit)) OR (ICU)) AND TITLE-ABS-KEY ((SARS-CoV-2) OR (COVID-19)))
Database: MEDLINE (via PubMed) Filters: last 3 years, English, Portuguese, Spanish Results: 50 Search strategy (14 April 2022) (((((burnout, professional[MeSH Terms]) OR (burnout[Title/Abstract])) OR (exhaustion[Title/Abstract])) AND ((((nurses[MeSH Terms]) OR (nurse[Title/Abstract])) OR (nurses[Title/Abstract])) OR (nurse practitioners[MeSH Terms]))) AND ((((intensive care units[MeSH Terms]) OR (intensive care units[Title/Abstract])) OR (intensive care unit[Title/Abstract])) OR (ICU[Title/Abstract]))) AND ((((SARS-CoV-2[MeSH Terms]) OR (SARS-CoV-2[Title/Abstract])) OR (COVID-19[MeSH Terms])) OR (COVID-19[Title/Abstract]))
Database: OPEN GREY Results: 5 Search strategy (14 April 2022) (burnout AND COVID-19)

**Table 2:** Data extraction form.

Scoping Review Details	
Scoping Review Title	The burnout of nurses in intensive care units and the impact of the pandemic of SARS-CoV-2: Protocol of a Scoping Review
Review Objective(s)	Map the scientific evidence related to the burnout of nurses in an intensive care unit and analyze the prevalence of this syndrome in these nurses during the COVID-19 pandemic
Review Question(s)	What is the impact of the pandemic of SARS-CoV-2 on the burnout of nurses in intensive care units? What is the prevalence of nurses’ burnout in intensive care units during the pandemic of SARS-CoV-2?
Inclusion/Exclusion Criteria	
Population	This review will consider studies that include nurses
Context	This review will consider studies addressing the burnout of nurses
Concept	This review will consider articles in which the context is inserted in the pandemic period of SARS-CoV-2 and in intensive care units
Types of Evidence Sources	This scoping review will consider any quantitative, qualitative and mixed methods study designs for inclusion. Additionally, systematic reviews will be considered for inclusion in the proposed scoping review
Evidence Source Details and Characteristics	
Author(s)	
Year of Publication	
Origin/Country of Origin (where the source was published or conducted)	
Aims/Purpose	
Population and Sample Size	
Details/Results Extracted from the Source of Evidence (concerning the concept of the scoping review)	
Level of the Burnout of Nurses	
Prevalence of Nurses’ Burnout During the Pandemic	

## Data Availability

Not applicable.
